# Clinician and Patient Factors Influencing Treatment Decisions: Ethnographic Study of Antibiotic Prescribing and Operative Procedures in Out-of-Hours and General Dental Practices

**DOI:** 10.3390/antibiotics9090575

**Published:** 2020-09-04

**Authors:** Wendy Thompson, Rosemary McEachan, Susan Pavitt, Gail Douglas, Marion Bowman, Jenny Boards, Jonathan Sandoe

**Affiliations:** 1 Division of Dentistry, University of Manchester, Manchester M13 9PL, UK; 2 Faculty of Medicine and Health, University of Leeds, Leeds LS2 9LU, UK; s.pavitt@leeds.ac.uk (S.P.); g.v.a.douglas@leeds.ac.uk (G.D.); M.C.Bowman@leeds.ac.uk (M.B.); J.A.Boards@leeds.ac.uk (J.B.); j.sandoe@leeds.ac.uk (J.S.); 3 Bradford Institute for Health Research, Bradford BD9 6RJ, UK; Rosie.McEachan@bthft.nhs.uk; 4 Faculties of Life Sciences & Health Studies, University of Bradford, Bradford BD7 1DP, UK

**Keywords:** antibiotic stewardship, behavioural influences, dental procedures, decision making, shared, motivation, primary health care, toothache

## Abstract

Operative treatment is indicated for most toothache/dental abscesses, yet antibiotics instead of procedures are often prescribed. This ethnographic study aimed to identify clinician and patient factors influencing urgent dental care for adults during actual appointments; and to identify elements sensitive to context. Appointments were observed in out-of-hours and general dental practices. Follow-up interviews took place with dentists, dental nurses, and patients. Dentist and patient factors were identified through thematic analysis of observation records and appointment/interview transcripts. Dentist factors were based on a published list of factors influencing antibiotic prescribing for adults with acute conditions across primary health care and presented within the Capability-Opportunity-Motivation-Behaviour model. Contextually sensitive elements were revealed by comparing the factors between settings. In total, thirty-one dentist factors and nineteen patient factors were identified. Beliefs about antibiotics, goals for the appointment and access to dental services were important for both dentists and patients. Dentist factors included beliefs about the lifetime impact of urgent dental procedures on patients. Patient factors included their communication and negotiation skills. Contextual elements included dentists’ concerns about inflicting pain on regular patients in general dental practice; and patients’ difficulties accessing care to complete temporary treatment provided out of hours. This improved understanding of factors influencing shared decisions about treatments presents significant opportunity for new, evidence-based, contextually sensitive antibiotic stewardship interventions.

## 1. Introduction

To prevent and slow the spread of infections resistant to antibiotics, the World Health Organization (WHO) has advised that antibiotics should only be prescribed when they are needed, according to current guidelines [1,2]. Recognizing the variety of contexts and challenges for dentistry across the globe, the Fédération Dentaire Internationale (FDI) World Dental Federation has advocated the development of national guidelines based on locally relevant evidence [3,4]. The FDI has highlighted different access to dentistry, different abilities of patients to pay for dental care, and different ways of people being able to access antibiotics as examples of contextual differences between high- and low–middle-income countries, which impact on the development of dental guidance [3,5]. Guidelines for treating acute dental conditions are generally based on the principle that operative dental procedures (extraction of a tooth or open drainage of its pulp chamber) are usually indicated to remove the cause of dental pain and/or infection; antibiotics are only required for a spreading infection [5,6,7,8,9]. Guidelines for the provision of antibiotics prior to dental treatment, such as prophylaxis for infective endocarditis, vary more widely in their details around the world [10,11,12]. Internationally, dentists are estimated to be responsible for one in ten of all antibiotics prescribed to humans [4,13] and rates of dental antibiotic overprescribing are high, with studies in the United States and United Kingdom demonstrating approximately 80% unnecessary use [14,15]. Across England’s publicly-funded National Health Service (NHS), the majority of dental antibiotics are prescribed for adult patients presenting with pain and/or acute infection in out-of-hours (OOH) or general dental practices (GDP) [16]. 

The need for new dental antibiotic stewardship (ABS) programmes and interventions designed with the context in mind and underpinned by behavioural science has been previously identified [17,18]. The Theoretical Domains Framework (TDF) is a theoretical lens through which to view the cognitive, affective, social and environmental influences on behaviour [19]. A published umbrella review of factors influencing decision making about antibiotic prescribing for adults with acute conditions across primary care and a systematic review in dentistry identified a list of 27 potentially-modifiable factors, each mapped to the TDF [20]. Two of these factors related to antibiotic prescribing as a substitute for a dental procedure, so understanding decision making about treatment options is important when designing new approaches to dental ABS. Few published studies have observed urgent dental treatment at the point of care, so a study of actual urgent dental appointments was recommended [20]. The significance of patient influence and paucity of evidence about shared decisions was also highlighted [20]. 

An ethnographic study provides a rich insight into people’s views, actions and environment, through detailed observations and interviews [21]. An ethnographic design enabled insight into dentist and patient factors influencing decision making about treatment in OOH and GDP settings, building on the published list of factors and their links to behavioural science [20]. To inform the development of new, evidence-based, theory-informed, contextually fit ABS programmes and interventions, this study had two aims: (1) to investigate what dentist and patient factors influence treatment decisions (including but not limited to antibiotic prescribing) for adults presenting with acute conditions to NHS England dental services; and (2) to compare how these factors differ between OOH and GDP contexts.

## 2. Results

These results were derived from analysis of the study data collected during the ethnographic observations and follow-up interviews. Exemplary quotes are presented to illustrate key findings. 

### 2.1. Description of Participants and Appointments

Between September 2017 and March 2018, 76 appointments were observed and/or audio-recorded involving 11 dentists (4 OOH and 7 GDP) across 9 research sites (2 OOH and 7 GDP). Between November 2017 and May 2018, follow-up interviews were completed with 28 key informants (13 patients, 10 dentists and 5 dental nurses). 

#### 2.1.1. Demographics of Participants

Demographic details of the dentists and patients who participated in the ethnographic observations and follow-up interviews are presented in Supplementary Material Tables S1 and S2. 

#### 2.1.2. Treatment Provided and Duration of Appointments

**Figure 1:**
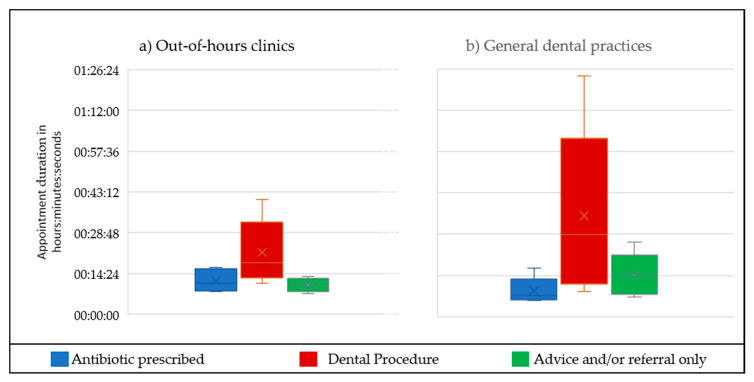
Summary of durations of the ethnographically observed appointments by treatment type and clinical setting: (**a**) out-of-hours and (**b**) general dental practice. When antibiotics were prescribed in addition to irrigation/dressing of a dry socket or pericoronitis (4/7 OOH appointments and 2/12 in GDP), the data have been included under ‘antibiotic prescribed’ rather than ‘dental procedure.’ No other dental procedures were provided in association with antibiotic prescribing. OOH, out of hours; GDP, general dental practices.

A summary of the treatment type (antibiotics, procedure, or advice/onward referral only) provided during the appointments is include in Supplementary Material Table S1. The duration of appointments by treatment type and setting (OOH vs. GDP) is provided in Figure 1. 

### 2.2. Dentist Factors Influencing Treatment Decisions

**Figure 2:**
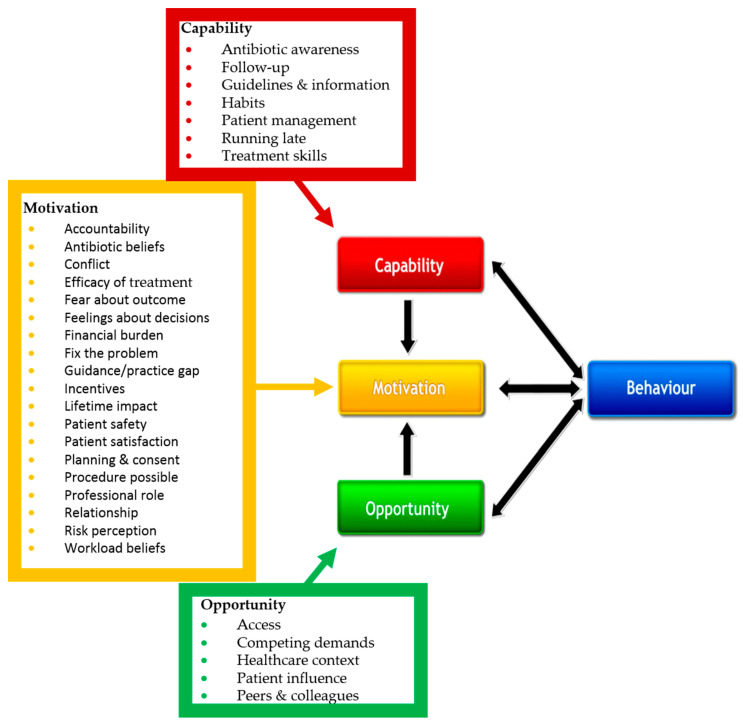
Dentist factors influencing treatment decisions whether to prescribe antibiotics for adult patients with acute conditions. Four factors were entirely new: ‘lifetime impact’, ‘running late’, ‘patient safety’ and ‘follow up’. The Capability-Opportunity-Motivation-Behaviour (COM-B) Model is reproduced under the terms of the Creative Commons Attribution Licence. [Adapted from Michie (2011)].

Thirty-one dentist factors were identified as influencing treatment during urgent dental appointments. A summary of the factors in the Capability-Opportunity-Motivation-Behaviour (COM-B) model is presented in Figure 2 and shows that over half of the factors (*n* = 19) relate to motivational aspects of behaviour. Further details about each factor associated with treatment decisions during this study are included in Supplementary Material Table S3. 

Of the thirty-one factors, twenty-seven were identified from the published list of factors associated with decisions about whether to prescribe antibiotics for adults with acute conditions across primary health care [20]. Access to the right care for the right patient at the right time, including routine and specialist care on referral, had been previously identified as an important factor for dentists. The ethnographic observations and interviews with dentists identified a new facet related to access: the sense of frustration experienced by dentists who were unable to support patients to access definitive care in primary or secondary care.

“The key factor behind today’s decision to prescribe antibiotics seemed to be the need to reduce swelling …this patient was anxious and didn’t have a regular GDP and was facing a denture. After the appointment, the dentist explained his frustration at the lack of options for patients like this one—not able to access NHS dental care—no one ‘taking on’ patients in [name of city]—possibly a 6 month waiting list leaving this patient with few options.”

*(Ethnographic observation—OOH)*

‘the dentist seemed angry about [the cost of dental treatment not covered by the NHS] on behalf of the patient’

*(Ethnographic observation—OOH)*

‘…he’s that scared that the fear of dentists overrides his pain … he’s in pain he’s got abscesses and there’s been an error or a mix up with his referral. Something like, he had been referred and they’d called him on the incorrect number, so he had failed to attend, so he had been taken off the referral list …. Oh, what do I do? I can’t refuse to give antibiotics if he’s in pain! But then how many antibiotics can we even prescribe?’

*(Interview—GDP Dentist)*

Five of these factors from the published list across primary health care had not previously been reported in dental studies. These dentist factors were: (1) ‘antibiotic awareness’ about potential adverse outcomes from antibiotic use, such as *Clostridioides difficile* infection; (2) ‘antibiotic beliefs’ about the risks of antibiotics and the importance of responsible use; (3) the degree of ‘accountability’ felt by dentists for their patterns of treatment/antibiotic prescribing; (4) concerns about ‘conflict’ which may result from failure to meet patient expectations for antibiotics; and (5) beliefs about the ‘financial burden’ which dental procedures place on patients and their ability to pay for definitive treatment to restore function and appearance. Further details of all thirty-one factors, including illustrative quotes from the dentist interviews, are provided in Supplementary Material Table S3.

An additional four dentist factors associated with treatment decisions were added to the list as their content did not fit with other factors already listed. Concerns about the ‘lifetime impact’ of dental procedures on patients, skills to deal with ‘running late’, identifying ‘patient safety’ as a goal for the appointment and the opportunity to ‘follow up’ patients after appointments are discussed in more detail below,

**Lifetime impact**—Concern about the impact on a patient’s quality of life resulting from an irreversible dental procedure (such as extraction of a problematic tooth) provided during an urgent dental appointment was expressed by most dentists. This related to the overall lifetime health and wellbeing of patients, such as their confidence and comfort when smiling, speaking, and eating. As explained by this OOH dentist during interview, anticipatory regret was a component which some dentists worried might be experienced by a patient once they were no longer in pain.

“When you are in a lot of pain, you want the pain to go and don’t think about future consequences. So I think I sometimes worry that patients when they are out of pain and once they have calmed down will then worry about this gap that possibly will not be able to be replaced because they don’t have a dentist.”

*(Interview—GDP Dentist)*

**Running late**—Most dentists reported running late for scheduled appointments to be a source of stress although their ability to cope with this stress was more varied. Some practices helped the dentists manage this stressor by ensuring dedicated slots each day for unscheduled patients. Some dentists reported being better able to deal with the inevitable late running caused by managing unscheduled patients.

“…because of the way the patients are usually squeezed in it’s a time management thing…I mean I get stressed when I’m running late generally anyway but you accommodate.”

*(Interview—GDP Dentist)*

A dental nurse provided more insight. Whilst talking about a time management approach sometimes used within her OOH clinic, they also highlighted the impact which running late can have on other patients.

“…sometimes [giving patients 5 to 10 minutes back in the waiting room] is needed … because you do have to think about the people that are waiting cos they do have appointments and they are often distressed too.”

*(Interview—OOH Dental Nurse)*

The prescribing of antibiotics rather than provision of a dental procedure was another approach to avoid running late shared by a dental nurse during interview.

“If someone’s been booked in literally for a five-minute appointment… [it] isn’t long enough [so the dentist] will give antibiotics instead.”

*(Interview—GDP Dental Nurse)*

**Patient safety**—When asked about their goal for urgent dental appointments, each dentist initially replied (using almost identical phraseology): ‘*to get them out of pain.’* Upon reflection, however, some dentists corrected their initial response to identify patient safety as their primary goal with resolution of symptoms a secondary goal.

“To get the patient out of pain. But actually, that’s a secondary goal of mine. My first goal is to make sure they’re not in danger…”

*(Interview—GDP Dentist)*

The critical safety aspect of urgent dental appointments and the potentially fatal risk associated with the failure of treatment to arrest the spread of dental infections, was highlighted by one dentist.

“You’re providing a service which, you know, is effectively saving lives…. Patients come in with a massive facial swelling, it could become quite severe and quite serious.’

*(Interview—OOH Dentist)*

Even dentists who did not explicitly recognize that patient safety was their primary goal were clearly aware of the risks associated with dental infections left to progress unhindered. In this example, the dentist views the risks of adverse events to antibiotics as less than the risk of a spreading dental infection.

“It’s very rare that I don’t manage to convince the patients to have it out …. [but if a patient declines care] just give them antibiotics just to keep them happy: (1) to prevent a complaint and (2) to prevent any sort of subsequent swelling or infection that may take them to A&E.”

*(Interview—OOH Dentist)*

**Follow up**—The ability to follow up patients provided feedback on outcomes to the dentist which enabled learning through experience. This feedback in GDP generally occurred at the following routine appointment with the patient as they only rarely returned or called to confirm that the urgent treatment had addressed the problem.

“I gave her the option to book in herself rather than book [a review] in proactively and since then I’ve not seen her, so I’m assuming it must’ve been fine.”

*(Interview—GDP Dentist)*

However, there is a strong potential for this factor to encourage learning about the success of treatment which is not in accordance with guidance. This included the use of antibiotics to manage patient satisfaction, irrespective of whether they were appropriate for the dental condition with which the patient was presenting.

“…managing the patient and managing the patient’s expectations as well as managing the tooth…”

*(Interview—GDP Dentist)*

One dentist candidly explained that they had made the decision some years ago to provide antibiotics rather than operative dental procedures (which are indicated by guidelines) for people with acute dental conditions, following reflection on her experience of patient dissatisfaction early in her career.

“My first and second year, I would try everything on the first appointment. But … after a few times I was still disappointed and making the patient unhappy. So, I decided to change the way I do it [using antibiotics] and it’s a lot better.”

*(Interview—GDP Dentist)*

### 2.3. Patient Factors Influencing Treatment Decisions

Nineteen patient factors were identified as influencing treatment during urgent dental appointments. The factors are listed in Table 1, with descriptions showing the wide-ranging issues incorporated. A key finding was revealed through triangulation of the results between the appointment transcripts and patient interviews: some patients strongly desired antibiotics.

“Is there no way I can just have antibiotics and come back after Christmas and have this done and have it removed? And can I be knocked out? Because honestly I’m terrified.”

*(Appointment transcript—OOH)*

“I wasn’t going to leave without any [antibiotics].”

*(Interview—OOH Patient)*

A more protracted negotiation between a patient and a dentist in GDP took place where the patient strongly desired antibiotics but the dentist (who had not previously treated this patient) declined to prescribe them, explaining they were not necessary:

Patient: *“… [I’ve] been given amoxicillin before and that’s been twice now…”*

Dentist: *“…there’s absolutely no indication to give you that.”*

Patient: *“I was actually given it last time I was here…”*

Dentist: *“…Antibiotics will make no difference in this case… So I can [list of all options]”*

Patient: *“Erm, I don’t really* want *that tooth out to be honest….”*

(Appointment transcript—GDP)

During follow-up interview, the patient explained his thoughts, feelings and approach which had influenced his observed actions the appointment.

*“I tried to push it. So I did say it quite a few times… giving the same reason that it did help me before…* [When the dentist declined to prescribe antibiotics] *I sort of knew at that point. I sort of felt disappointed because of the fact that them saying no it means that* [the tooth] *was in a bad state and* [antibiotics] *wouldn’t help at all. So, I did feel disappointed … It was all leaning towards the tooth being taken out.*”

(Interview—GDP Patient)

### 2.4. Contextual Elements Affecting Treatment Decisions—Comparing OOH vs. GDP Settings

Contextual elements were identified relating to seventeen dentist factors (as detailed in Table 1) and one patient factor (‘access’). 

Concern about the possibility of inflicting pain on patients when providing dental procedures (an element of the ‘fear about outcomes’ dentist factor) was expressed by some dentists in GDP but none in OOH:

“My worst nightmare is that the anesthetic is not working and anything you touch, or try to go in the pulp, that the patient feels it.”

(Interview—GDP Dentist)

Conversely, the time required to gain valid consent for dental procedures (an element of the ‘workload’ dentist factor) was highlighted by some working in OOH but not GDP. The issue related to the context of OOH as patients had not usually been treated at the clinic before and no health care records were available. The stages of the process of consent took longer, including building rapport with patients, taking a full medical history, diagnosing the condition, discussing the relative risks and benefits of treatment options and ensuring sufficient information and time for patients to decide which treatment (if any) to accept. With urgent dental appointments during this study typically lasting approximately 16 min (see Figure 1), the minutes spent whilst patients consider their options were reported as a source of frustration by some dentists:

“Sometimes when patients can’t decide, they’re sat there thinking ‘Oh I don’t know what to do. I might ring this person. I might ring that person…’ It does become quite frustrating …in the back of your mind, ‘Oh g*d, we’ve not got much time left.’”

*(Interview—OOH Dentist)*

For patients recruited from OOH clinics, access routine primary dental care to complete temporary work undertaken in the OOH clinic (or for routine preventative care into the future) affected their decisions about urgent dental treatment.

“I need [a dentist…the OOH dentist] put clean inside—after 10 days everything is out and painful… I am waiting [to find a dentist to finish the treatment].”

*(Interview—OOH patient)*

For both patients recruited from GDP and dentists working in GDP, access to secondary care services (such as treatment of a dental-phobic patient under sedation) was a problem which influenced urgent treatment decisions:

“He just needs an extraction and, bless him, he’s that scared that the fear of dentists overrides his pain. [Now its infected again I had to] prescribe a further course of antibiotics [and called about] the referral to explain the situation to try and get him up the list faster.”

*(Interview—GDP Dentist)*

## 3. Discussion

Thirty-one factors which were associated with dentists’ treatment decisions for patients with acute conditions were identified during this ethnographic study, of which nine had not previously been reported in dental studies. The dentist factors have been presented within the COM-B model to facilitate their use by practitioners and researchers seeking to develop new evidence-based, theory-informed ABS programmes and interventions. Whilst traditionally ABS interventions have targeted the ‘cognition of individual clinicians’ (capability within the COM-B model) or the ‘microculture of individual clinical units’ (environmental context within the COM-B model) [22], we found most of the dentist factors mapped to motivational aspects of behaviour. Nineteen factors which affected patients in relation to treatment during urgent dental appointments were identified, of which access and antibiotic beliefs were also dentist factors. Seventeen contextual elements were identified by comparing OOH and GDP settings. Understanding of these factors and contextual elements presents significant opportunities for the design of new evidence-based, theory-informed approaches to tackle unnecessary dental antibiotic prescribing and improve the quality of care for adults with acute dental conditions. The findings also highlight that dental ABS may fail if the clinical context is not considered.

Ethnography has previously been used to explore antibiotic prescribing decision making in secondary care, comparing acute medical and acute surgical contexts [23]. A key strength of ethnographic studies is the depth of insight provided; a key weakness is the narrowness of their focus which limits their generalizability [24]. Generalizability was addressed in this study by building on a list of factors published in an umbrella review across primary health care settings and systematic review in dentistry [20]. Narrowness of focus was addressed by employing a maximum variation sampling strategy to recruit dentists from across a range of settings, based on characteristics identified as important in the published systematic review in dentistry. Concerns inherent to ethnographic research include observation bias (the Hawthorne Effect [25]) and selection bias. The authors considered that these might lead to observed urgent dental care being provided more in accordance with guidance and hence the results could be an underestimation of their impact in unobserved dental care.

Poor access to the right care for the right patient at the right time has been previously identified as a factor which influences dental antibiotic prescribing decisions [26,27]. We found access to primary and secondary care dental services to be a significant issue influencing dentists and patients in their decision making in both OOH and GDP settings. As responsibility for addressing access to services lies beyond the scope of conventional practice-based ABS interventions, strategic consideration should be given to issues of access to services, including by those responsible for delivering National Action Plans for combatting antibiotic resistance [28]. 

Beliefs about the risks of antibiotics have previously been identified as a key influence on decisions by primary care medics and community pharmacists [20,29]. Our ethnographic study confirmed ’antibiotic beliefs’ as influencing dentists too and identified it as a patient factor also. Interestingly, our study and another about antibiotics for upper respiratory tract infections [30] found that patients reported a good understanding the risks (and lack of benefits) of antibiotics, they still wanted them. The Wellcome Trust has also recognized the significance of patient beliefs about antibiotic risks and has recommended introducing a sense of personal jeopardy to messaging [31]. Whilst fear-based messaging is generally avoided in public health campaigns, it has been shown to be successful for reducing unnecessary antibiotic use when combined with empowering messages for patients about how to manage without antibiotics [32]. These are important aspects for future ABS interventions and public health messaging. 

The strong feelings expressed during interviews by some dentists and some patients for antibiotics surprised the authors. It has previously been suggested that scant evidence exists that patients demand antibiotics or pressure clinicians for them [33]. This ethnographic study showed some patients strongly desire antibiotics and some use their well-developed negotiation skills to put dentists under pressure to receive them. Further research to understand the tipping point at which different people will start to avoid rather than desire antibiotics should provide useful insight to assist the development of interventions across health care, not just within dentistry.

Recognizing the influence of context on the success of quality improvement interventions is crucial to designing, implementing and successfully replicating them in new settings [34]. Understanding the context of urgent dental care in OOH and GDP is key to the design and implementation of dental ABS programmes and interventions. Approximately 30% of people in England see a dentist only when they have a dental problem [35] and 15% of people in the United States last attended a dentist because of pain [36]. Dentists in OOH see people with acute dental problems as a matter of course and rarely see each patient more than once [26]. Conversely, dentists in GDP see patients regularly to help them maintain their oral health and avoid the need for urgent care. The importance of this trusting, enduring relationship and the associated emotional attachment between dentist and patient through continuity of care in GDP was highlighted by both dentists and patients. Furthermore, the viability of general dental practices (which are generally run as private businesses) is reliant on repeat business and nurturing a long-term professional relationship with patients is desirable [37,38]. Dentists in GDP understandably expressed concern about inflicting pain on patients as this is inconsistent with maintaining relations. Dentists in OOH were less concerned about this, which in turn made decisions to provide operative procedures easier.

An ethnographic study of risk work in GDP has found that the process of consent requires getting patients to accept risks and take responsibility to make a decision [39]. Lack of previous relationship with patients in OOH together with lack of access to patient medical records contributed to additional pressure on time when gaining valid consent [40] before dental procedures in OOH. Lack of access to patient records and time pressures in OOH medical clinics were also identified in a qualitative study of antibiotic decision making for patients with respiratory tract infections [41]. The impact of time pressures increasing the likelihood of providing antibiotics rather than a dental procedure has been widely reported [15,27,42]. Whilst this study was designed for maximum variation sampling rather than to enable statistical analysis, simple descriptive analysis of the appointments ethnographically observed provides some indication that antibiotic prescribing takes less time than providing a dental procedure (see Figure 1). Quantitative research to understand how long is long enough for urgent dental appointments is recommended to enable service standards to be underpinned with real-world evidence.

## 4. Materials and Methods 

This research involved human participants and was conducted in accordance with the Declaration of Helsinki. Ethical approval for this study was gained in two stages. University of Leeds Dental Research Ethics Committee (DREC ref: 120416/WT/202 dated 27/10/2016) covered identification of potential research sites. Bradford/Leeds Research Ethics Committee (REC:16/YH/0487 dated 9 February 2017 and updated 12 May 2017) covered all other aspects. 

Practice management teams at each research site were trained to become local research staff, responsible for initial approach, recruitment and consent of patients to this study. Informed consent was obtained from dentists, dental nurses and patients before data collection.

### 4.1. Sampling and Data Collection 

#### 4.1.1. Ethnographic Observation Sample 

This study was undertaken in OOH and GDPs providing care for patients of NHS England Lancashire and West Yorkshire Area Teams. Maximum variation sampling of sites and purposive sampling of dentists were based on characteristics of practices and dentists identified in a systematic review of factors associated with antibiotic prescribing for adults with acute dental conditions [20] (see Supplementary Material Table S4). Convenience sampling of dental nurses working with participating dentists and adults attending urgent dental appointments was employed. Full details of the sampling criteria for patients are in Supplementary Material Table S5.

Practices were invited to express interest in participation via email. During initial practice visits to potential research sites, dentists and dental nurses were provided with information leaflets and invited to participate. Practice management teams at each research site were trained to become local research staff, responsible for initial approach, recruitment and consent of patients to this study. Informed consent was obtained from dentists, dental nurses and patients before data collection.

#### 4.1.2. Interview Sample 

All dentists and dental nurses who participated in the ethnographic observations were invited for follow-up telephone interview. Maximum variation sampling of patients for interview was based on a systematic review of factors associated with antibiotic prescribing for adults with acute dental conditions [20] (details are in Supplementary Material Table S6). No more than two patients per dentist were interviewed to mitigate the risk of identifying patterns of behaviour during the research which might warrant overriding the principle of confidentiality and anonymity. Interviews with key informants continued until no new dentist factors, patient factors or differences between OOH and GDP contexts emerged, indicating saturation had been achieved.

### 4.2. Data Collection Methods

#### 4.2.1. Ethnographic Observations

The ethnographic study design included direct observation and/or audio-recording of urgent dental appointments and follow-up telephone interviews. Additional data were collected from patients before their appointments and from dentists and dental nurses immediately after each appointment to assist selection of cases for follow-up interview in accordance with the sampling criteria (see Supplementary Material Figures S1 and S2, respectively). All appointments were audio-recorded, transcribed verbatim and anonymized; the duration of each appointment was noted. In addition, some OOH appointments were directly observed by a non-clinical member of the research team (JB or MB) using an observation record form (see Supplementary Material Figure S3). 

#### 4.2.2. Interviews 

Telephone interviews with the patients, dentists and dental nurses were undertaken by the lead researcher/author (WT). The interviews were semi-structured using interview schedules which had been tailored for each case based on issues identified from the audio-recording transcripts and questionnaires completed by the dentists and dental nurses following each appointment. (see Supplementary Material Figure S4). All interviews were audio-recorded, transcribed verbatim and anonymized.

#### 4.2.3. Data Synthesis and Interpretation 

Deductive thematic analysis of the appointment and interview transcripts and observation record forms [43] used the published list of factors [20]. Additional factors were added to the list as necessary during the analysis. Each new factor was mapped to the TDF [19] to align with the other factors in the published list, and then linked via the TDF domain to the Capability-Opportunity-Motivation-Behaviour (COM-B) [44] model. COM-B is an intuitive model of behaviour change and its use aimed to facilitate use of the results by researchers designing and/or practitioners implementing ABS interventions. Inductive thematic analysis of the patient interview transcripts was undertaken to identify patient factors associated with treatment decisions during urgent dental appointments. Comparison of the dentist and patient factors between OOH and GDP was undertaken to identify contextual elements. 

Coding of all observation record forms and transcripts was undertaken by researcher WT using NVivo_11_Plus software. Researcher RRCM coded 10% of the transcripts independently. Discrepancies or disagreements were resolved through discussion. Analysis of the interview transcripts continued until the factors and contextual elements reached saturation (i.e., no new dentist or patient factors or contextual elements emerged).

Sense checks of the factors and contextual elements were provided by other members of the research/author team (GVAD, JATS and SHP) and this study’s patient-public involvement and engagement contributors.

## 5. Conclusions

Decision making about treatment during urgent dental appointments is multifaceted and factors vary from person to person and between contexts. Thirty-one dentist factors and nineteen patient factors were identified in this ethnographic study of OOH and GDP in NHS England. Some dentists continue to prescribe antibiotics for reasons other than clinical need and some patients continue to ask for antibiotics. Some dentists in general dental practice are concerned about inflicting pain on their regular patients. Some patients attending out-of-hours dental clinics experience difficulties accessing care to complete temporary treatment, which results in them entering a cycle of repeat attendance for temporary treatment. A “one size fits all” approach to antibiotic stewardship is, therefore, unlikely to be successful. 

Most of the dentist factors identified related to motivational influences, such as beliefs about antibiotics and concerns about the lifetime impact of dental procedures provided during urgent dental appointments. Access to primary and secondary care dental services was found to be an important environmental factor impacting on both dentists and patients. Significant opportunities exist for the design of new evidence-based, theory-informed contextually fit approaches to tackle unnecessary dental antibiotic prescribing and improve the quality of care for adults with acute dental conditions.

## Figures and Tables

**Table 1:** Patient factors influencing treatment decisions during urgent dental appointments.

Patient Factor	Description
Access	Ease of access to routine or specialist dental care, such as referral for sedation.
Antibiotic beliefs	Patient expectation or beliefs about the need for, or ability of, antibiotics to treat acute dental conditions. Some saw antibiotics as a quick fix whilst others expressed concern about the risks of antibiotics.
Antibiotic goal	Goal of appointment was to obtain antibiotics. Some patients stated that they had a plan as to how they would obtain them.
Beliefs about procedures	Patient expectation or beliefs about dental procedures, such as extraction or endodontic (root canal) treatment.
Communication/negotiation	Patient communication/negotiation skills in relation to dental appointments, including patient recognition of constraints within which the dentist was working.
Costs	Views about cost effectiveness and affordability of dental care (including for private care such as implants to fill spaces left by urgent procedures).
Delaying tactic	Aim to delay losing a tooth or extensive dental work until no other options were possible.
Emotional attachment	Degree of emotional attachment a patient has to their teeth impacted on their willingness to agree to extraction.
Engagement in consent	Willingness or ability of the patient to engage in the process of consent and sharing decisions, including capacity to take in, weigh up and use information.
Family, friends and colleagues	Influence from family, friends and colleagues about appropriate treatment or legitimacy of time off work for dental problems.
Fear about outcome	Fear that the condition might become worse or cause a more severe problem.
Feelings about dentistry	Feelings such as anxiety, phobia, or desire with respect to urgent dental care. Some wished to avoid procedures/aesthetic injections, while others desired antibiotics.
Information and advice	Sources of information and advice about dental conditions and treatment options used by patients, including the internet.
Medicines knowledge	Knowledge about antibiotics and analgesics for dental conditions, including the risks and benefits of antibiotics for individuals and society.
Minimize disruption	Goal of appointment was to minimize disruption to their routine, such as reducing time off work by fixing the problem quickly.
Previous experience	Previous experience of treatment for a similar condition, such as antibiotics for pain.
Pain relief goal	Goal of appointment was to stop pain.
Trust	Degree of trust in the dental team.
Understanding the condition	Patient’s understanding of the causes of acute condition/symptoms

## Supplementary Materials

The following are available online at https://www.mdpi.com/2079-6382/9/9/575/s1, Figure S1: Case report form ‘Registration information’ for data collection from patients immediately before each appointment. Figure S2: Short questionnaires for data collection from a) dentists and b) dental nurses immediately after each appointment. Figure S3: Extract from Observation Record Form. Figure S4: Extract from a tailored interview schedule for dentists, Table S1: Summary of data relating to patients observed and their urgent appointments. Table S2: Summary of data relating to the 21 patients selected for interview (and their appointments characteristics) by sampling criteria. Table S3: Summary of the 31 potentially modifiable dentist factors associated with treatment decisions during urgent dental appointments. Table S4: Sampling matrix for research sites and dentists. Table S5: Sampling criteria to guide recruitment of patients to ethnographic observations. Table S6: Sampling criteria for patients to interview.
