# Supplementary material for: Clinician and Patient Factors Influencing Treatment Decisions: Ethnographic Study of Antibiotic Prescribing and Operative Procedures in Out-of-Hours and General Dental Practices
Source: Antibiotics (Basel). 2020 Sep 4;9(9):575. doi: 10.3390/antibiotics9090575 (PMC7558392; doi:10.3390/antibiotics9090575)
Supplement: Supplementary file 1 [file antibiotics-09-00575-s001.pdf]

Supplementary Material for:

Dentist- and patient-factors influencing treatment decisions: Ethnographic study of antibiotic prescribing and operative procedures in out-of-hours and general dental practices.

Table S1 Summary of data relating to patients observed and their urgent appointments

Characteristic	Measure	Out-of-hours dental clinic (n=33)	General dental practice (n=43)
Patient gender	Male	21	18
	Female	12	25
Patient ethnicity	White British	22	36
	Other	11	7
Level of patient's educational attainment	No formal qualifications	7	7
	Level 1 or 2 (GCSE or equivalent)	9	9
	Level 3 or 4 (A' Level or	6	4
	equivalent)	5	9
	Level 5 or 6 (College/University)	3	4
	Level 7 or 8 (Postgraduate)	3	10
Level of deprivation of the patient's home area	High (IMD 1-3)	21	20
	Medium (IMD 4-6)	9	13
	Low (IMD 7-10)	3	10
Regularly attends dental check-ups	Yes	0	31
	No	33	12
Presence of accompanying adult	Yes	5	7
	No	28	36
Dental anxiety of patient (nurse view)	Seems anxious (score 3-5)	9	10
	Seems not anxious (1-2)	24	33
Treatment provided	Antibiotics	7	12
	Other	26	31
Pressure felt by dentist to provide a particular treatment	Yes	4	6
	No	29	37
Dentist's confidence in the diagnosis (self reported)	Not certain (score 1-3)	4	11
	Certain (score 4-5)	29	32
Appointment long enough? (dentist view)	Yes	24	35
	No/Not sure	9	8
Level of clinician stress	Dentist:		
	Not stressful (score 1-2)	35	21
	Stressful (score 3-5)	8	12
	Nurse:		
	Not stressful (score 1-2)	31	34
	Stressful (score 3-5)	2	9

Table S2 Summary of data relating to the 21 patients selected for interview (and their appointments characteristics) by sampling criteria. After setting a date for the interviews, 8 of the patients dropped out leaving a total of 13 patients (8 GDP and 5 OOH) who were interviewed.

Characteristic	Measure	Out-of-hours dental clinic (n=8)	General dental practice (n=13)
Patient gender	Male	3	8
	Female	5	5
Patient ethnicity	White British	5	9
	Other	3	4
Level of patient's educational attainment	No formal qualifications	2	2
	Level 1 or 2 (GCSE or equivalent)	2	2
	Level 3 or 4 (A' Level or	3	2
	equivalent)	1	4
	Level 5 or 6 (College/University)	0	2
	Level 7 or 8 (Postgraduate)	0	1
Level of deprivation of the patient's home area	High (IMD 1-3)	5	4
	Medium (IMD 4-6)	2	6
	Low (IMD 7-10)	1	3
Regularly attends dental check-ups	Yes	0	12
	No	8	1
Presence of accompanying adult	Yes	3	2
	No	5	11
Dental anxiety of patient (nurse view)	Seems anxious (score 3-5)	4	2
	Seems not anxious (1-2)	4	11
Treatment provided	Antibiotics	4	6
	Extraction	0	3
	Open & dress pulp	3	1
	Other treatment	1	1
	Advice/referral only	0	2
Pressure felt by dentist to provide a particular treatment	Yes	3	3
	No	5	10
Dentist's confidence in the diagnosis (self reported)	Not certain (score 1-3)	2	6
	Certain (score 4-5)	6	7
Appointment long enough? (dentist view)	Yes	4	12
	No/Not sure	4	1
Level of clinician stress	Dentist:		
	Not stressful (score 1-2)	5	9
	Stressful (score 3-5)	3	4
	Nurse:		
	Not stressful (score 1-2)	7	11
	Stressful (score 3-5)	1	2

Table S3 Summary of the 31 potentially modifiable dentist-factors associated with treatment decisions during urgent dental appointments. New factors found during this study are highlighted. Factors associated with antibiotic prescribing for adults with acute conditions from an umbrella review across primary care and a systematic review in dentistry[1] are reproduced by permission of Oxford University Press.

Dentist factor	Description	TDF Domain
Access	Access to the right care for the right patient at the right time, including routine and specialist care (e.g. sedation) on referral. Availability of an accurate medical history. Provision of treatment over more than one appointment. Continuity of care during working hours and OOH (e.g. weekends) and/or by a single dentist. Access whilst the patient is on holiday and for patients who live remotely.	Environmental context & resources
Accountability*	Dentist feels like they are held accountable for their individual treatment patterns.	Beliefs about consequences
Antibiotic awareness*	Knowledge about the relationship between antibiotic use and adverse outcomes such as resistant infections.	Knowledge
Antibiotic beliefs*	Personal responsibility towards antibiotics, including the belief that antibiotics are low risk and blaming others for their misuse.	Beliefs about consequences
Competing demands	Priority given to ensuring sufficient time to treat unscheduled patients in accordance with guidelines and other clinical requirements e.g. record keeping. Practice/clinic approach to dealing with urgent appointments (e.g. daily slots of defined length kept free, ability of team to flex time to meet need, squeezing patients in between routine patients etc). Impact of running late (e.g. due to unscheduled patients or the dentist arriving late for the clinic). Workload impact on competing demands due to shortage of dentists and dental nurses associated with staff turnover, illness, maternity or annual leave.	Environmental context & resources
Conflict*	Fear of conflict with patient due to dissatisfaction and concern about subsequent loss of the patient to the practice.	Emotion
Efficacy of treatment	Beliefs about the efficacy of different treatment options, including: ability of antibiotics versus other approach/procedure to resolve conditions.	Beliefs about consequences
Fear of outcome	Fear about adverse outcomes, including anxiety about making a mistake and the prospect of serious complications if patients with symptoms go without antibiotics.	Emotion
Feedback loops**	Follow-up of patients after an urgent appointment, or as part of continuing care, thus facilitating dental team learning through feedback about outcomes. N.B. If a patient returns in pain this approach may encourage prescribing rather than treating according to guidance.	Behavioural regulation
Feelings about decisions	Feeling about the appointment and decisions, including frustration at lack of consent for appropriate treatment and	Emotion

	positive or negative emotions related to delivering treatment.	
Financial burden*	Beliefs about financial burden on patients, including ability to pay for the definitive treatment plan to restore oral function and appearance.	Beliefs about consequences
Fix the problem	Goal for urgent dental appointments is to fix the patient's problem: symptomatic relief and/or preventing the problem returning.	Goals
Guidance-practice gap	Gap between guidance and clinical practice, including dentists' concerns about the application of national guidelines to specific clinical encounters and belief about whether their clinical practice (such as delayed prescribing) adheres to relevant dental guidance.	Beliefs about consequences
Guidelines & information	Knowledge about relevant guidelines and where they source other relevant information (including the internet and social media) about appropriate treatment for acute dental conditions and dental prescribing.	Knowledge
Habits	Habits of dentists in relation to patterns of treatment and/or prescribing.	Memory, attention & decision processes
Healthcare context	Healthcare system context in relation to the delivery of urgent dental care, including drug prescribing/dispensing processes and availability of antibiotics from outside of the healthcare system.	Environmental context & resources
Incentives	Incentives for and against certain treatment options, including the impact of a '*time is money*' business approach on unscheduled/urgent appointments and the financial risk of losing dissatisfied patients.	Reinforcement
Life-time impact**	Belief about the consequences that providing a dental procedure (such as extraction of the problematic tooth) will have on the patient's overall lifetime health and wellbeing (such as their confidence smiling, speaking and eating).	Beliefs about consequences
Patient influence	Influence of patients including: dentist's perceptions about patient expectations/demands for particular treatment types e.g. antibiotics; sharing decision making and obtaining valid consent for operative dental procedures; and dealing with patients who frequently fail to attend routine appointments.	Social influences
Patient management	Skills in patient management, diagnosis, treatment planning and consent, including eliciting concerns, interpreting the patient's description of their symptoms, managing anxious patients, managing expectations, managing uncertainty and avoiding confrontation. Negotiation, persuasion and education. Communication skills, including using images (radiographs & photographs) and test results as communication tools.	Skills
Patient safety**	Patient safety identified as a goal of urgent dental care.	Goals
Peers & colleagues	Influence of peers and other colleagues, relating to: dental nurse & reception influence (including time management);	Social influences

	relationship with dentists or doctors treating the same patient (including professional courtesy to avoid encroaching / territorialism); different treatment options offered by different dentist; and utility of peer support/second opinions when dealing with difficult situations.	
Planning & consent	Belief about ability to plan treatment and gain consent during urgent appointments, including 'do nothing' options and managing anxious/phobic patients.	Beliefs about capabilities
Procedure possible	Dentist's belief about whether it is possible to provide treatment (due to issues other than relating to their skillset) during urgent appointments, including the ability to achieve adequate local anaesthesia, manage a non-vital tooth without local anaesthesia and/or to provide operative treatment (in accordance with guidelines) to dentally phobic patients without sedation.	Beliefs about capabilities
Professional role	Influence of professional role on managing urgent appointments, including: what is means to care for patients; and feeling 'morally obliged' to offer something tangible (to 'do nothing' is difficult).	Professional role & identity
Relationship	Desire to build/maintain a good dentist-patient relationship.	Goals
Risk perception	Beliefs about risks when managing the patient's condition, including: pain during or after provision of a procedure; failure of (or inability to complete) an operative procedure; worsening of the condition; or medicolegal complaint.	Beliefs about consequences
Running late**	Skills to cope with the impact of over running appointments, including time management, team working and resilience.	Skills
Treatment skills	Skills in providing urgent procedures, including placing local anaesthetic by injection in difficult clinical situations or lancing an abscess in the presence of swelling.	Skills
Workload	Belief about impact on workload, including time taken to: diagnose/explain/gain informed consent/deliver treatment options or refer to a specialist service.	Beliefs about consequences

Key:

[1] Thompson, W.; Douglas, G.; Pavitt, S.; Sandoe, J.; McEachan, R.; Tonkin-Crine, S. Factors associated with prescribing of systemic antibacterial drugs to adult patients in urgent primary health care, especially dentistry. *J. Antimicrob. Chemother.* **2019**, *74*, 2139–2152

***** Factors from the published list across primary healthcare which not previously identified from dental studies

****** New factors added to the list as the themes did not fit with factors from the published list across primary healthcare.

Table S4 Sampling matrix for research sites and dentists.

Sample matrix	Criteria	Source
**Out-of-hours and general dental practice research sites**		
Geographic location	Rural vs Urban	Rural Urban Classification (UKOffice of National Statistics) 'Predominantly urban' through 'Predominantly rural'[1]
Provider type	Independent<br>Corporate<br>Social enterprise	Care Quality Commission healthcare provider website.[2]
**General dental practice research sites only**		
Rate of antibiotic prescribing	High vs Low rate	Upper vs lower quartile by rate of antibiotics dispensed per practice across Lancashire and West Yorkshire.[3]
Size of practice patient base	At least 1000 adult patients	NHS Digital 'Patients seen over a 24-month period 1 April 14 to 31 March 16'.[4]
**Dentists**		
Gender	Male vs Female	
Where trained	UK vs Overseas	
When trained	Before 2001*<br>During or After 2001	

* UK dental antibiotic prescribing guidance was first published in the UK in 2000

[1]https://assets.publishing.service.gov.uk/government/uploads/system/uploads/attachment_data/file/539241/Guide_to_applying_the_rural_urban_classification_to_data.pdf

[2] www.CQC.co.uk

[3]Unpublished data

[4] https://digital.nhs.uk/data-and-information/publications/statistical/nhs-dental-statistics/nhs-dental-statistics-for-england-2015-16

Table S5 Sampling criteria to guide recruitment of patients to ethnographic observations.

Inclusion criteria	Exclusion criteria
**Aged 18 years and over.**	Severe/debilitating pain or distress which prevents their ability to consent to participate
**Attending for NHS urgent dental care to address: 'dental or facial pain not controlled by over-the-counter drugs' or 'acute dental or soft tissue infection'.**	Attending for routine NHS or any private dental care.
**Willing to have his/her appointment observed and/or audio recorded.**	Accompanied during the appointment by someone under the age of 18 years.
**Able and willing to give their informed consent to participate.**	Accompanied during the appointment by someone who was not able or willing to consent to participate.
	If needed by the patient, no translation service available

Table S6 Sampling criteria for patients to interview.

Characteristic	Criteria	Source
**Patient gender**	Male vs Female	Case Report Form
**Patient ethnicity**	White British vs Other	
**Level of patient's educational attainment**	No formal qualifications<br>Level 1 or 2 (GCSE or equivalent)<br>Level 3 or 4 (A' Level or equivalent)<br>Level 5 or 6 (College/University)<br>Level 7 or 8 (Postgraduate)	
**Level of deprivation of the patient (based on postcode of residence)[1]**	Indices of Multiple Deprivation<br>High-level (IMD 1-3)<br>Low-level (IMD 7-10)	
**Regularly attends dental check-ups at the practice**	Yes vs No	
**Accompanying by another adult in the appointment**	Yes vs No	
**Treatment/care provided**	Antibiotics vs Other (extraction, Open & Dress Pulp, Other Treatment, No Treatment)	Dentist Questionnaire
**Pressure felt by dentist to provide a particular treatment**		
**Level of confidence in the diagnosis**	Certain vs Not Certain	
**Appointment was long enough**	Yes vs No	Dentist and Dental Nurse Questionnaire
**Level of clinician stress**	Stressful vs Not Stressful	
**Anxiety of patient as assessed by dental nurse**	Seemed to be anxious vs Seemed not to be anxious	Dental Nurse Questionnaire

[1] http://imd-by-postcode.opendatacommunities.org/imd/2019

Figure S1 Case report form 'Registration information' for data collection from patients immediately before each appointment.

Patient's gender ☐ Male ☐ Female

Patient's ethnicity

☐ *White British* ☐ *Other white background*

☐ *Asian or Asian British* ☐ *Black or Black British*

☐ *Chinese* ☐ *Mixed background*

☐ *Any other ethnic group* ☐ *Patient declined*

What was the last (highest) educational qualification gained by the patient?

☐ *GCSE or equivalent* ☐ *A' Level or equivalent*

☐ *College/university degree or equivalent* ☐ *Postgraduate degree or equivalent*

☐ *No formal qualifications* ☐ *Unclear/ patient decline*

Patient's postcode ☐☐☐☐ ☐☐☐☐

Patient pays NHS fees ☐ Yes ☐ No ☐ Don't know

Patient attends regular dental check-ups at this dental practice/clinic ☐ Yes ☐ No ☐ Don't know

If no, are they attending for urgent care only? ☐ Yes ☐ No ☐ N/A

Figure S2 Short questionnaires for data collection from a) dentists and b) dental nurses immediately after each appointment.

QUESTIONNAIRE FOR DENTISTS

Which factors in dentist-patient interactions influence treatment in urgent dental care?

Case Identifier: __ __ / __ __

1. **How confident were you of the diagnosis for this patient?** (Please circle one option)

   Not very certain 1 2 3 4 5 Absolutely certain

2. **Did you feel pressure from anyone about the type of treatment provided today?** (Please circle one option)

   Yes No Not sure

   If 'Yes', please give details..

   ....

3. **What treatment did you provide today?** (Please circle ALL that apply)

   Antibiotics Extraction Open & dress pulp Other treatment No treatment

   If 'Other' or 'No' treatment, please give details...

   ....

4. **Was the scheduled appointment slot long enough?** (Please circle one option)

   Yes No Not sure

   If 'No', what happened as a result?...

   ....

5. **How stressful did you find the appointment?** (Please circle one option)

   Not at stressful 1 2 3 4 5 Extremely stressful

   Please give details...

   ....

6. **Was there anything else about this appointment which you feel would be useful for us to consider in our study about what influences decision making?** (Please circle one option)

   Yes No Not sure

   If 'Yes', please explain ....

QUESTIONNAIRE FOR DENTAL NURSES

Which factors in dentist-patient interactions influence treatment in urgent dental care?

Case Identifier: __ __ / __ __

1. **Was the scheduled appointment slot long enough?** (Please circle one option)

   Yes No Not sure

   If 'No', what happened as a result?...

   ....

   ....

2. **How anxious did the patient appear?** (Please circle one option)

   Not all anxious 1 2 3 4 5 Extremely anxious

   Please give details...

   ....

3. **How stressful did you find the appointment?** (Please circle one option)

   Not at all stressful 1 2 3 4 5 Extremely stressful

   Please give details...

   ....

4. **Was there anything else about this appointment which you feel would be useful for us to consider in our study about what influences decision making?** (Please circle one option)

   Yes No Not sure

   If 'Yes', please explain ....

Figure S3 Extract from Observation Record Form.

TREATMENT PROVIDED

10. If operative treatment was performed, when did the nurse start preparing the equipment/materials?

No operative treatment | Before consent process started | After consent gained

11. If a prescription was given today, who completed most of the prescription form?

No prescription given | Dentist | Dental Nurse | Not sure

12. If any advice leaflets were given today, what were they about?

Operative treatment options | Pain relief | Antibiotics | Not sure

Comment:

Figure S4 Extract from a tailored interview schedule for dentists.

Topic 2 – Views on providing urgent dental care

- Thank you – looking generally at urgent dental care appointments, what is your personal goal for urgent dental appointments?

  Prompt: Do you have a system in place which could be used to monitor whether you achieve it? Do you ever recall patients for treatment after urgent care? Or make courtesy follow-up phone calls?

- Next I'm interested more specifically about the two appointments selected for follow-up. Have you had a chance to remind yourself about them in advance of this phone call? Yes / No

- Tell me about the appointment with {Insert name of patient}

  Prompt: Uncertainty 5 Treatment Abs Pressure No Stress 1 Other other dentist – different advice – referral to specialist care [illegible]

- Tell me about the appointment with {Insert name of patient}

  Prompt: Uncertainty 5 Treatment open + dress Pressure No Stress (3) why? Other Not long enough language barrier Access to primary care
